# Characteristics and safety outcomes of rheumatoid arthritis patients treated with AMGEVITA^®^ in the United Kingdom using data from a national register

**DOI:** 10.1186/s13075-026-03807-9

**Published:** 2026-04-21

**Authors:** Argentina E. Servin, Mark Lillie, Ran Jin, Laura C. Coates, Michele Brunori, Latha Krishnamoorthy, Neil Accortt, Frances Humby

**Affiliations:** 1 Center for Observational Research (CfOR), Amgen Inc., Thousand Oaks, CA USA; 2 Amgen Ltd., Cambridge, United Kingdom; 3 Nuffield Department of Orthopaedics, Rheumatology and Musculoskeletal Sciences, University of Oxford, Oxford, UK; 4 Global Medical Affairs, Amgen Switzerland AG, Rotkreuz, Switzerland; 5 Queen Mary University of London, London, UK; 6 Department of Rheumatology, Guy’s and St Thomas’ NHS Trust and King’s College London, London, UK

**Keywords:** Rheumatoid arthritis, Biosimilars, Biologics, BSRBR-RA

## Abstract

**Background:**

Over the past decade, rheumatoid arthritis (RA) management in the UK has evolved with increased use of biologic and targeted synthetic Disease-Modifying Anti-Rheumatic Drugs (DMARDs). The introduction of biosimilars, including AMGEVITA^®^, has expanded treatment options and improved access for patients. This study aims to (1) describe the clinical characteristics and serious adverse events (SAEs) in RA patients treated with AMGEVITA^®^, and (2) similarly, describe the incidence of these outcomes from the British Society for Rheumatology Biologics Register for Rheumatoid Arthritis (BSRBR-RA) anti-tumor necrosis factor (anti-TNF) comparison cohort.

**Methods:**

The BSRBR-RA is a national registry for patients with RA receiving biologic treatments, including anti-TNF inhibitors (e.g., adalimumab) and non-anti-TNF biologics, designed to evaluate the long-term safety and effectiveness of these agents in clinical practice. SAE analyses derived from an interim pharmacovigilance report on a subset of all AMGEVITA^®^ and anti-TNF patients in the BSRBR-RA based on the Manchester Template Report. Eligible participants were ≥ 16 years of age with RA who registered in the registry within 6 months of initiating AMGEVITA^®^. Descriptive statistics were calculated for all variables of interest.

**Results:**

From 01/01/2019 to 31/03/2022, 288 RA patients initiating AMGEVITA^®^ were enrolled. Follow-up ended 30/11/2023. The mean (SD) age was 59.7 (12.0) years; 86 (28.0%) were male; and mean (SD) disease duration at initiation was 9.9 (9.8) years. Two-thirds of AMGEVITA^®^ patients were biologically naïve and 9.8% were using steroids at initiation. The crude incidence rate of SAEs in the AMGEVITA^®^ cohort was 9.6 per 1,000 patient-years (95% CI: 3.1–22.4), including 37.5 serious infections per 1,000 patient-years (95% CI: 95% CI: 23.2–57.3). For context, the anti-TNF comparator cohort had a crude SAE rate of 13.3 per 1,000 patient-years (95% CI: 10.6–16.5) and a serious infection rate of 49.1 per 1,000 patient-years (95% CI: 43.8–54.8).

**Conclusion:**

The crude incidence rates of SAEs and serious infections among AMGEVITA^®^ users were within the range observed for the anti-TNF comparator cohort. These findings provide supportive real-world safety data, although differences in baseline characteristics and the absence of adjusted analyses should be considered when interpreting results.

## Background

Rheumatoid arthritis (RA) is a chronic inflammatory condition that can lead to joint damage and reduced quality of life. RA is a chronic autoimmune disease marked primarily by inflammatory joint symptoms, including swelling, pain, and stiffness, with additional systemic manifestations such as fatigue and increased acute-phase reactants (ESR, CRP) observed in many patients [1]. Central to the pathogenesis of RA is tumor necrosis factor α (TNFα), a pro-inflammatory cytokine that drives synovial inflammation and joint damage. TNFα stimulates the production of matrix metalloproteinases (MMPs), particularly MMP-3, which degrade extracellular matrix components, while suppressing the activity of tissue inhibitors of metalloproteinases (TIMPs) [2–5]. Additionally, TNFα promotes osteoclastogenesis, contributing to bone erosion.

Over the past decade, the management of RA in the United Kingdom (UK) has advanced considerably with the continued integration of biologic disease-modifying antirheumatic drugs (bDMARDs) and the more recent adoption of targeted synthetic DMARDs (tsDMARDs), such as Janus kinase (JAK) inhibitors into standard therapeutic algorithms [6]. Among the bDMARDs, tumour necrosis factor inhibitors (TNFi) remain a foundational treatment class, with adalimumab as the most widely prescribed advanced therapeutic. Since the patent expiry of the originator product (Humira) in 2018, several biosimilar adalimumab products have entered the UK market. Notably, AMGEVITA^®^ (adalimumab biosimilar by Amgen) was among the first to be launched and has played a significant role in NHS efforts to enhance treatment access [7, 8]. Regulatory approval was based on a comprehensive body of evidence demonstrating analytical, pharmacokinetic, and clinical equivalence to Humira [9–12].

Despite strong regulatory endorsement and robust evidence of clinical comparability, the uptake of biosimilars in UK rheumatology practice has been uneven. Differences among regions in how biosimilars are commissioned and funded, along with variations in clinicians’ prescribing preferences and patients’ comfort with transitioning from originator biologics, have all contributed to this inconsistent adoption. As a result, real-world data remain essential for assessing the long-term safety and effectiveness of biosimilars across diverse patient populations treated in clinical practice. To address this need, the present study was conducted as part of a post-authorization safety (PASS) commitment for AMGEVITA^®^. Utilizing the British Society for Rheumatology Biologics Register for Rheumatoid Arthritis (BSRBR-RA)—a well-established registry designed to monitor the long-term safety of biologic and biosimilar therapies in routine clinical practice—this study aims to (1) describe the clinical characteristics and serious adverse events (SAEs) in RA patients treated with AMGEVITA^®^, and (2) similarly, describe the incidence of these outcomes from the British Society for Rheumatology Biologics Register for Rheumatoid Arthritis (BSRBR-RA) anti-tumor necrosis factor (anti-TNF) comparison cohort.

## Methods

### Study design

This analysis was conducted as part of Amgen’s PASS commitment to monitor the safety of AMGEVITA^®^ in routine clinical practice using data from the BSRBR-RA. The study is required under the EU Risk Management Plan (Category 3) for AMGEVITA^®^ and was agreed with the European Medicines Agency. It is registered in the EU PAS catalogue (EUPAS31796) with an actual start date of 15 November 2019 and a planned final report in October 2027 [13]. The objective was to describe the safety profile of AMGEVITA^®^ among patients with RA in a real-world setting, rather than to perform formal comparative analyses between treatment cohorts.

### Data source

The British Society for Rheumatology (BSR) established a nationwide register for patients aged 16 years and older with RA receiving biologic treatments, known as the British Society for Rheumatology Biologics Register for Rheumatoid Arthritis (BSRBR-RA) [14]. This register is designed as a national, prospective study with the primary aim of evaluating the long-term toxicity of these agents in routine clinical practice. A key feature of the BSRBR-RA register is the inclusion of a comparison group of patients with active rheumatoid arthritis who are being treated with conventional disease-modifying agents. Additionally, the data allow for assessment of the benefits of biologic treatments in relation to their potential toxicity and adverse events.

### Study population

These analyses include BSRBR-RA enrolled patients with RA who initiated AMGEVITA^®^ between 2019 and 2022 (enrollment period) as well as other RA patients who initiated anti-TNFs (adalimumab [excluding AMGEVITA^®^], etanercept, and infliximab) between 2010 and 2023. The overall study is scheduled for 8 years (2019–2027). Here, we report here on data collected through November 2023, using the Manchester Template Report for the safety outcomes (SAEs) and the annual data set for the non-safety analyses.

*Anti-TNF comparator cohort*: Eligible patients were ≥ 16 years of age, diagnosed with RA, newly treated (i.e. within 6 months of registration) with originator adalimumab, etanercept, or infliximab as their first b/tsDMARD. In accordance with the BSRBR-RA protocol, patients enrolled in the anti-TNF cohort were biologic-naïve at registration, reflecting the registry’s original design to capture first-line biologic users for long-term safety monitoring [15, 16]. All patients provided voluntary informed consent (or consent from a legal guardian, if applicable). These agents were selected because they represent the longest-standing anti-TNF therapies within the BRSBR-RA, each with extensive mature safety follow-up in the registry. This ensured the availability of stable and well-characterized reference data to provide contextual benchmarks for crude incidence rates, rather than to enable direct comparison. Other anti-TNF agents (e.g., golimumab and certolizumab pegol) were not included becausetheir cumulative follow-up time and sample sizes were comparatively limited within the BSRBR-RA study period, resulting in unstable crude incidence rate estimates for contextual benchmarking.

Patients exited the study upon death, withdrawal of consent, or completion of the follow-up period.

*AMGEVITA*^*®*^* cohort*: Eligible patients were ≥ 16 years of age or older, diagnosed with RA, registered in the BSRBR-RA within 6 months of initiating AMGEVITA^®^. All participants (or their legal guardian, if applicable) provided voluntary informed consent. In contrast to the original anti-TNF cohort, which enrolled only biologic-naïve patients, the AMGEVITA^®^ cohort includes both biologic-naïve and biologic-experienced individuals, reflecting contemporary clinical practice and NHS commissioning policies that support both initiation and non-medical transitioning to biosimilars [17, 18]. This broader inclusion captures a real-world mix of patients initiating AMGEVITA^®^ either as their first biologic or following prior biologic therapy. Patients exited the study upon discontinuation of AMGEVITA^®^, death, withdrawal of consent, or completion of the follow-up period.

### Outcome measures

The primary outcome measure was incidence of serious infections, defined as serious infectious events requiring intravenous antibiotics or hospitalization, as well as the incidence of serious hypersensitivity reactions.

Serious infections were identified using multiple complementary sources within the BSRBR-RA [15, 19]. At baseline and at 6-monthly intervals for the first 3 years of follow-up (annually thereafter), treating rheumatologists completed standardized case report forms that included specific questions regarding adverse events, including hospital admissions, infections, malignancies, and deaths. To enhance case capture, participants were also sent postal questionnaires at 6-monthly intervals to report hospitalizations, infections, and other health events; reported events were subsequently verified with the treating clinical team. In addition, linkage was performed with national administrative datasets. In England, Hospital Episode Statistics (HES) provided independent records of inpatient admissions, including those coded as infections using ICD-10. Mortality data were obtained from the Office for National Statistics (ONS), with cause of death reviewed for infectious contributions. All suspected cases were reviewed and validated by registry staff, and events were classified using standardized definitions. This multimodal approach—combining clinician report, patient self-report, and administrative data linkage—was designed to maximize completeness and accuracy in capturing the incidence of serious infections and hypersensitivity reactions.

The secondary outcome measure was incidence of other SAEs, including cardiac, hematologic, malignant events, and death. SAEs were defined according to standard regulatory criteria as events that were fatal, life-threatening, required inpatient hospitalization or prolongation of existing hospitalization, or resulted in persistent or significant disability or incapacity or were otherwise deemed medically important by the treating physician. Event capture mirrored that for serious infections, drawing on rheumatologist case report forms, patient questionnaires, and linkage with national datasets (Hospital Episode Statistics and the Office for National Statistics), with all events validated by registry staff.

### Exposure variables

Exposure was defined from the index date (date of first dose of AMGEVITA® or comparator anti-TNF) until treatment discontinuation, death, last follow-up, or end of study (November 2023), whichever occurred first. For safety outcomes other than malignancy, patients were considered at risk only while on their index therapy, and follow-up ended at discontinuation. For mortality analyses, a 90-day risk window after discontinuation was applied, consistent with prior registry practice [15, 19]. Malignancy outcomes were captured irrespective of treatment status, with follow-up continuing until loss to follow-up or the end of data collection.

### Other covariates

Among the AMGEVITA®, both the primary and secondary outcome measures were stratified by prior advanced therapeutic use and biologic status: biologic-naïve (no biologic exposure prior to initiating AMGEVITA®) or biologic-experienced (prior use of adalimumab or different anti-TNF). Other covariates include demographic (e.g., age, sex, ethnicity, lifestyle factors) and clinical characteristics (RA history, disease activity and severity, previous and current treatments, comorbidities and medical history) that are part of BSRBR-RA case report form (CRF).

### Data analysis

Incidence of primary and secondary outcomes (i.e., serious infections, hypersensitivity reactions, and other serious adverse events [safety concerns]) were estimated as cumulative incidence rates (events per 1,000 patient years exposure) with 95% confidence intervals (95% CI) based on the exposure definitions above. For context, incidence rates were also calculated for the BSRBR-RA anti-TNF comparison cohort. No formal comparisons were conducted and no statistical adjustments for baseline differences were performed. Person-time was accrued from the index date until treatment discontinuation, death, last follow-up, or end of study, whichever occurred first. Importantly, patients were not censored at the time of their first adverse event; instead, follow-up continued until censoring events as defined above, allowing for multiple events per patient within and across categories. This approach is consistent with registry-based pharmacovigilance methodology and explains why the denominator of person-years is consistent across event categories, with the numerator reflecting the number of events observed [17].

The anti-TNF cohort has been followed in the registry for a substantially longer period than the AMGEVITA^®^ cohort, reflecting the earlier introduction of these agents and the registry’s longitudinal design rather than any analytical decision. Expressing incidence rates per 1,000 patient-years accounts for differences in total follow-up time; however, longer observation periods may increase the likelihood of capturing recurrent or late-onset events and can reflect temporal changes in clinical practice. This methodological approach ensures comprehensive event capture while allowing meaningful contextualization of crude rates between cohorts.

Frequency and percentages (with 95% CI where appropriate) were provided for categorical variables. Summary statistics for continuous variables included the number of patients, mean, median, SD or standard error, minimum, and maximum. Significance tests included *t*-tests for continuous variables and chi-square test for categorical variables. Missing data were treated as not reported, and no imputations were performed. The extent of missingness varied by variable; for example, BMI was not available for 42.0% of AMGEVITA^®^ patients and 25.8% of anti-TNF comparator patients, whereas most demographic and clinical variables had low levels of missingness (< 10%). Because this was a descriptive pharmacovigilance analysis based on aggregate registry data, no adjusted modeling was conducted, and therefore imputation of baseline covariates was not undertaken.

## Results

From January 1, 2019 to March 31, 2022, 288 RA patients initiating AMGEVITA^®^ were enrolled in this study. Among the anti-TNF comparator cohort (n = 2,229), the mean (SD) age of the population was 57.2 (12.9) years, 75.7% of patients identified as female and mean (SD) disease duration at initiation was 8.9 (9.3) years. Majority of patients in the anti-TNF cohort (94.6%) had a history of methotrexate use and prior corticosteroid use was reported among 77.7% of patients. Consistent with the BSRBR-RA protocol design, > 99% of anti-TNF comparator patients were biologic-naïve at registration. Two patients (< 1%) had prior biologic exposure recorded, likely reflecting historical reporting discrepancies rather than protocol deviation.” Among the AMGEVITA^®^ cohort (*n* = 288), the mean (SD) age of the population was 59.5 (12.0) years, 70.8% of patients identified as female and mean (SD) disease duration at initiation was 9.4 (9.6) years. Furthermore, 86.8% had a history of methotrexate use and 32.7% reported prior corticosteroid use. In contrast, a higher proportion of AMGEVITA^®^ users had previously received biologic therapies (29.5%). The AMGEVITA^®^ cohort contributed 560.4 person-years of follow-up, while the anti-TNF comparator cohort contributed 6,419.8 person-years, corresponding to mean follow-up durations of approximately 2 and 3 years, respectively. Additional patient baseline (i.e., at enrolment and prior to initiation) characteristics for both AMGEVITA^®^ and anti-TNF patients are presented in Table 1.

**Table 1:** Patient baseline characteristics

CHARACTERISTICS	AMGEVITA^®^ patients (*N* = 288)	Anti-TNF patients (*N* = 2,229)	*P*-value
Gender			
Male n (%)	84 (29.2%)	542 (24.3%)	0.0731
Female, n (%)	204 (70.8%)	1, 687 (75.7%)	
Age			
Mean (SD)	59.5 (12.0)	57.2 (12.9)	0.0041
Duration of disease			
Mean (SD)	9.4 (9.6)	8.9 (9.3)	0.8606
Number of previous csDMARDs			
None reported	43 (14.9%)	90 (4.0%)	< 0.0001
1	125 (43.4%)	843 (37.8%)	
2	88 (30.6%)	880 (39.5%)	
3+	32 (11.1%)	416 (18.6%)	
Prior use of methotrexate			
Yes	250 (86.8%)	2,109 (94.6%)	< 0.0001
No	38 (13.2%)	120 (5.4%)	
Prior use of steroids^a^			
Yes	74 (32.7%)	1,726 (77.7%)	< 0.0001
No	214 (74.3%)	503 (22.6%)	
Prior use of biologics^a^			
Yes	85 (29.5%)	2 (0.1%)	< 0.0001
No	203 (70.5%)	2,215 (99.4%)	
Smoking status			
Current	40 (13.9%)	384 (17.2%)	0.2612
Former	92 (31.9%)	815 (36.6%)	
Never	113 (39.2%)	827 (37.1%)	
Tender joint count			
Mean (SD)	9.7 (7.5)	14.5 (7.2)	< 0.0001
Swollen joint count			
Mean (SD)	5.5 (4.7)	8.5 (5.1)	< 0.0001
Global Health VAS (mm)			
Mean (SD)	69.5 (25.5)	73.3 (20.1)	0.0073
ESR			
Mean (SD)	25.5 (21.5)	30.3 (24.8)	0.0200
CRP			
Mean (SD)	16.8 (23.5)	21.9 (28.0)	0.0127
DAS28			
Mean (SD)	5.0 (1.5)	5.9 (1.1)	< 0.0001
Any comorbidity reported			
Yes	174 (60.4%)	1,361 (61.1%)	0.8335
No	114 (39.6%)	868 (38.9%)	
BMI, by category			
<18.5	2 (0.7%)	38 (1.7%)	0.4227
18.5-<25	52 (18.1%)	506 (22.7%)	
25-<30	61 (21.2%)	505 (22.7%)	
30-<35	30 (10.4%)	341 (15.3%)	
35+	22 (7.6%)	264 (11.8%)	
Not available	121 (42.0%)	575 (25.8%)	

^a^Prior biologic use” and “prior steroid use” were coded according to BSRBR-RA data dictionary definitions as “history of” exposure, indicating any previous use before registry registration rather than medication use restricted to the baseline visit

Patients in both the AMGEVITA^®^ and anti-TNF cohorts exhibited moderate disease activity approximately six months after initiation, with a mean DAS28 score ranging from 3.4 to 3.7 (*p* = 0.1276). Similarly, both AMGEVITA^®^ and anti-TNF cohorts reported a mean tender joint count of 3.8–5.2 (*p* = 0.0128), consistent with moderate disease activity, while the mean swollen joint count remained 2.0-2.4 (*p* = 0.1888), indicative of low disease activity. Additional clinical measures among AMGEVITA^®^ and anti-TNF patients are detailed in Table 2.

**Table 2:** Clinical measures among AMGEVITA^®^ and Anti-TNF patients 6-months after initiation

	AMGEVITA^®^ patients	Anti-TNF patients	*P*-value
	*N* = 288	*N* = 2,229	
DAS28			
Mean (SD)	3.4 (1.4)	3.7 (2.3)	0.1276
Tender joint count			
Mean (SD)	3.8 (5.3)	5.2 (6.5)	0.0128
Swollen joint count			
Mean (SD)	2.0 (3.0)	2.4 (3.5)	0.1888
ESR			
Mean (SD)	17.3 (14.4)	19.7 (19.7)	0.3137
CRP			
Mean (SD)	9.3 (16.2)	10.4 (17.4)	0.5241
Global Health VAS (mm)			
Mean (SD)	48.2 (26.3)	40.4 (26.5)	0.0012

Among patients initiating AMGEVITA^®^, notable differences in baseline characteristics were observed between those with and without prior biologic experience. Patients who were biologic-experienced (*n* = 85) had a longer mean disease duration (14.7 vs. 7.3 years) and lower disease activity at baseline, with mean DAS28 scores of 3.6 compared to 5.6 (*p* = < 0.0001) in those who were biologic-naïve. This group also presented with fewer tender and swollen joints (4.1 vs. 11.9 [*p* = < 0.0001] and 2.0 vs. 6.9 [*p* = < 0.0001], respectively), lower inflammatory markers (CRP: 9.6 vs. 20.2 mg/L [*p* = 0.0023]; ESR: 19.8 vs. 28.6 mm/h [*p* = 0.0150]), and better patient-reported global health (VAS: 50.3 vs. 77.3 mm; *p* = < 0.0001). Additionally, a higher proportion of patients who were biologic-experienced had no prior use of csDMARDs (28.2% vs. 9.4%; *p* = 0.0007) and had a greater burden of comorbidity, with 18.8% reporting three or more comorbidities compared to 7.4% in the biologic-naïve group. Gender distribution and BMI were similar across groups (Table 3).

**Table 3:** Patient baseline characteristics for AMGEVITA^®^ patients, stratified by prior biologic use

	AMGEVITA^®^ patients biologic-experienced	AMGEVITA^®^ patients biologic-naïve	*P*-value
	*N* = 85	*N* = 203	
Gender			0.9528
Male - n (%)	25 (29.4%)	59 (29.1%)	
Female - n (%)	60 (70.6%)	144 (70.9%)	
Age			0.0391
Mean (SD)	61.7 (12.3)	58.5 (11.8)	
Duration of disease			< 0.0001
Mean (SD)	14.7 (11.0)	7.3 (8.1)	
Number of previous csDMARDs			0.0007
None reported	24 (28.2%)	19 (9.4%)	
1	32 (37.6%)	93 (45.8%)	
2	20 (23.5%)	68 (33.5%)	
3+	9 (10.6%)	23 (11.4%)	
Tender joint count			< 0.0001
Mean (SD)	4.1 (4.7)	11.9 (7.2)	
Swollen joint count			< 0.0001
Mean (SD)	2.0 (3.4)	6.9 (4.4)	
Global Health VAS (mm)			< 0.0001
Mean (SD)	50.3 (27.7)	77.3 (19.9)	
ESR			0.0150
Mean (SD)	19.8 (18.8)	28.6 (22.4)	
CRP			0.0023
Mean (SD)	9.6 (15.0)	20.2 (25.9)	
DAS28			< 0.0001
Mean (SD)	3.6 (1.5)	5.6 (1.1)	
Number of comorbidities			0.0380
None	29 (34.1%)	85 (41.9%)	
1	27 (31.8%)	67 (33.0%)	
2	13 (15.3%)	36 (17.7%)	
3+	16 (18.8%)	15 (7.4%)	
BMI			0.3164
Mean (SD)	29.2 (7.1)	28.1 (6.0)	

*As percentage of patients with prior RA medication history available

Among the anti-TNF comparator cohort, the incidence rate of serious infections was 49.1 events per 1,000 person-years. (95% CI: 43.8–54.8) and the incidence rate was 13.3 SAEs events per 1,000 person-years. In the AMGEVITA^®^ cohort, the incidence rate of serious infections was 37.5 events per 1,000 person-years. (95% CI: 23.2–57.3) and the incidence rate of SAEs was 9.6 events per 1,000 person-years. Among SAEs reported in the AMGEVITA^®^ cohort, the most frequently reported were non-opportunistic serious infections (*n* = 11), malignant events (*n* = 9), and CNS disorders (*n* = 6) over the same 4,487 person-years of follow-up respectively. Venous thromboembolic events were infrequent, with no events reported among AMGEVITA^®^ users during the 90-day exposure window and 12 events (1.9 per 1,000 person-years) in the anti-TNF cohort. Additional reported SAEs for the AMGEVITA^®^ and anti-TNF patients are listed in Table 4.

**Table 4:** Non-Adjusted incident rates per 1,000 patient-years of follow-up calculated for Serious adverse events (SAEs)

Event	AMGEVITA^®^ (*N* = 288)			Anti-TNFs (*N* = 2,229)		
	Events	Person-years follow-up	Incident rate, per 1000 PYs (95% CI)	Events	Person-years follow-up	Incident rate, per 1000 PYs (95% CI)
Total Serious Infection	21	560.4	37.5 (23.2, 57.3)	315	6419.8	49.1 (43.8, 54.8)
Pneumonia	6	560.4	10.7 (3.9, 23.3)	139	6419.8	21.7 (18.2, 25.6)
* Non-opportunistic*	6	560.4	*10.7 (3.9*,* 23.3)*	133	6419.8	*20.7 (17.3*,* 24.6)*
* Opportunistic*	0	560.4	*0.0 (0.0*,* 6.6)*	6	6419.8	*0.9 (0.3*,* 2.0)*
Septicaemia	1	560.4	1.8 (0.0, 9.9)	14	6419.8	2.2 (1.2, 3.7)
* Non-opportunistic*	1	560.4	*1.8 (0.0*,* 9.9)*	14	6419.8	*2.2 (1.2*,* 3.7)*
* Opportunistic*	0	560.4	*0.0 (0.0*,* 6.6)*	0	6419.8	*0.0 (0.0*,* 0.6)*
Bone/Joint infection	3	560.4	5.4 (1.1, 15.6)	15	6419.8	2.3 (1.3, 3.9)
* Non-opportunistic*	3	560.4	*5.4 (1.1*,* 15.6)*	15	6419.8	*2.3 (1.3*,* 3.9)*
* Opportunistic*	0	560.4	*0.0 (0.0*,* 6.6)*	0	6419.8	*0.0 (0.0*,* 0.6)*
Herpes Zoster	0	560.4	0.0 (0.0, 6.6)	0	6419.8	0.0 (0.0, 0.6)
* Non-opportunistic*	0	560.4	*0.0 (0.0*,* 6.6)*	0	6419.8	*0.0 (0.0*,* 0.6)*
* Opportunistic*	0	560.4	*0.0 (0.0*,* 6.6)*	0	6419.8	*0.0 (0.0*,* 0.6)*
Other serious infection	11	560.4	19.6 (9.8, 35.1)	147	6419.8	22.9 (19.3, 26.9)
* Non-opportunistic*	11	560.4	19.6 (9.8, 35.1)	143	6419.8	22.3 (18.8, 26.2
* Opportunistic*	0	560.4	*0.0 (0.0*,* 6.6)*	4	6419.8	0.6 (0.2, 1.6)
TB	0	560.4	*0.0 (0.0*,* 6.6)*	2	6419.8	0.3 (0.0, 1.1)
Total cardiac disorders	5	560.4	8.9 (2.9, 20.8)	79	6419.8	12.3 (9.7, 15.3)
CHF (new or worsening)	0	560.4	0.0 (0.0, 6.6)	9	6419.8	1.4 (0.6, 2.7)
Myocardial infarction	1	560.4	1.8 (0.0, 9.9)	18	6419.8	2.8 (1.7, 4.4)
Other cardiac events	4	560.4	7.1 (1.9, 18.3)	52	6419.8	8.1 (6.0, 10.6)
CNS Disorders	6	560.4	10.7 (3.9, 23.3)	54	6419.8	8.4 (6.3, 11.0)
Demyelination	0	560.4	0.0 (0.0, 6.6)	1	6419.8	0.2 (0.0, 0.9)
Peripheral neuropathy	1	560.4	1.8 (0.0, 9.9)	1	6419.8	0.2 (0.0, 0.9)
Other CNS	5	560.4	8.9 (2.9, 20.8)	52	6419.8	8.1 (6.0, 10.6)
Total haematologic events	1	560.4	1.8 (0.0, 9.9)	28	6419.8	4.4 (2.9, 6.3)
Aplastic anaemia	0	560.4	0.0 (0.0, 6.6)	0	6419.8	0.0 (0.0, 0.6)
Pancytopaenia	0	560.4	0.0 (0.0, 6.6)	2	6419.8	0.3 (0.0, 1.1)
Agranulocytosis	0	560.4	0.0 (0.0, 6.6)	2	6419.8	0.3 (0.0, 1.1)
Other dyscrasia	1	560.4	1.8 (0.0, 9.9)	24	6419.8	3.7 (2.4, 5.6)
Total malignant events	9	1081.2	8.3 (3.8, 15.8)	217	18738.3	11.6 (10.1, 13.2)
Lymphoproliferative	2	1081.2	1.8 (0.2, 6.7)	12	18738.3	0.6 (0.3, 1.1)
*Lymphoma (NHL*,* Hodgkins)*	1	1081.2	*0.9 (0.0*,* 5.2)*	8	18738.3	*0.4 (0.2*,* 0.8)*
* Myeloma*	0	1081.2	*0.0 (0.0*,* 3.4)*	1	18738.3	*0.1 (0.0*,* 0.3)*
* Leukaemia*	1	1081.2	*0.9 (0.0*,* 5.2)*	3	18738.3	*0.2 (0.0*,* 0.5)*
Non-melanoma Skin Cancer	1	1081.2	0.9 (0.0, 5.2)	63	18738.3	3.4 (2.6, 4.3)
Other malignant solid tumours	6	1081.2	5.5 (2.0, 12.1)	142	18738.3	7.6 (6.4, 8.9)
* Of which*,* brain neoplasms*	0	1081.2	*0.0 (0.0*,* 3.4)*	1	18738.3	*0.1 (0.0*,* 0.3)*
* Of which*,* glioblastoma*	0	1081.2	*0.0 (0.0*,* 3.4)*	1	18738.3	*0.1 (0.0*,* 0.3)*
Death (90-day exposure)	1	603.9	1.7 (0.0, 9.2)	58	6576.4	10.6 (9.2, 12.2)
VTE	0	560.4	0.0 (0.0, 6.6)	12	6419.8	1.9 (1.0, 3.3)

## Discussion

This study is being conducted as part of a PASS commitment with the European Medicines Agency for AMGEVITA^®^. It provides post-authorization real-world evidence on the safety profile of the adalimumab biosimilar AMGEVITA^®^ in patients with rheumatoid RA using data from the BSRBR-RA. Although formal comparative analyses were not performed, the crude incidence rates of SAEs among AMGEVITA^®^ users were consistent within the range observed in patients receiving other anti-TNF therapies within the registry. The inclusion of long-established anti-TNF agents (adalimumab, etanercept, and infliximab) as a reference cohort was intended to provide stable, well-characterized registry data for contextualizing crude incidence rates, rather than to enable formal comparative analyses.

The demographic characteristics of the cohort indicate a mean age of 59.7 years, with a relatively small proportion of male patients (28.0%), consistent with the known female predominance in RA. The mean disease duration at treatment initiation was approximately 9.9 years, suggesting that patients had long-standing RA before initiating AMGEVITA^®^. Although many patients in our cohort had long disease duration, registry data from the BSRBR-RA indicate that about two-thirds of patients starting AMGEVITA^®^ in the UK were biologic-naïve, while the remainder had previously received other biologic therapies, including originator adalimumab (Humira) [17]. This pattern likely reflects both evolving clinical practice and NHS England’s commissioning framework, which encourages the use of biosimilars to improve cost-effectiveness while maintaining clinical outcomes. As a result, AMGEVITA^®^ may be used as a first biologic in some patients or as a switch from an originator biologic in others. NHS guidance issued in 2018 supported the transition to biosimilar adalimumab for patients already established on originator therapy, provided clinical equivalence and patient engagement were ensured [20]. Such guidance-driven transitions may have occurred in patients whose RA was already well controlled, meaning AMGEVITA^®^ may have been initiated in the context of low disease activity, partially explaining the lower mean DAS observed at initiation. Even among biologic-naïve patients, the mean time from RA diagnosis to AMGEVITA^®^ initiation exceeded seven years. Part of this delay could reflect a calendar effect, as many patients in the BSRBR-RA were diagnosed several years before AMGEVITA^®^ became available in the UK, inherently increasing the diagnosis-to-initiation interval. These findings align with prior studies showing that access barriers, regional prescribing variation, and patient or physician preferences continue to influence treatment timing [21].

Additionally, in the AMGEVITA^®^ cohort, patients with prior biologic exposure had a substantially longer disease duration at baseline (median: 13 years vs. 4 years) and presented with lower baseline disease activity, as reflected by lower Global Health VAS scores, ESR, CRP, and DAS28 values, compared with biologic-naïve patients. These differences likely reflect treatment history and accumulated disease management.

Furthermore, the high proportion of biologic-naïve patients initiating AMGEVITA^®^ underscores increasing confidence in biosimilars as first-line options in RA management. This trend has important implications for clinical practice and health policy, as broader adoption of biosimilars may support earlier access to effective therapies and maintain high standards of care without compromising clinical outcomes [22, 23]. Historical National Institute for Health and Care Excellence (NICE) guidance, which initially required a DAS28 score of ≥ 5.2 for biologic initiation in treatment-naïve patients, may have contributed to earlier discrepancies in treatment eligibility [24, 25].

Differences in prior medication use between the AMGEVITA^®^ and anti-TNF comparator cohorts have important implications for interpreting treatment outcomes and real-world biosimilar utilization. Prior methotrexate use was similar among both cohorts (86.8% vs. 94.6%), but corticosteroid exposure was lower in the AMGEVITA^®^ group (32.7% vs. 77.7%), potentially reflecting evolving prescribing practices, reduced dependence on corticosteroids, or differences in patient comorbidity profiles. The higher prevalence of biologic-experienced patients in the AMGEVITA^®^ cohort (29.5% vs. 0.1%) aligns with registry design, as the anti-TNF comparator cohort included only biologic-naïve participants. This distinction also reflects the treatment landscape during the period of biosimilar introduction, when non-medical transitioning from the originator adalimumab (Humira) to biosimilars was strongly encouraged by national commissioning frameworks and formulary policies in the UK and Europe [26, 27]. Collectively, these findings emphasize the importance of accounting for treatment history and prescribing context when interpreting real-world biosimilar data.

The most frequently observed SAEs in the AMGEVITA^®^ cohort included non-opportunistic serious infections, malignant events, and central nervous system disorders, over 4,487 person-years of follow-up. Venous thromboembolic events were infrequent across both cohorts, with no events reported among AMGEVITA^®^ users during the 90-day exposure window and 12 events in the anti-TNF cohort. These findings, while informative, may also be a result of the shorter follow-up time and underlying population differences in the AMGEVITA^®^ cohort, and therefore, should be evaluated in light of those limitations. Clinical trials and post- marketing surveillance have demonstrated similar rates of adverse events, including infections and injection site reactions, between AMGEVITA^®^ and its reference product [6, 28]. Regulatory reviews by the European Medicines Agency (EMA) concluded that AMGEVITA^®^ exhibited no clinically meaningful differences in safety, efficacy, or immunogenicity compared to adalimumab, supporting its approval across the European Union and the UK [29]. Real-world data from European RA cohorts have further reinforced the safety equivalence, with no new safety signals observed after transitioning to or initiating treatment with AMGEVITA^®^ [30–32].

This study has several limitations. It was designed as a descriptive, pharmacovigilance analysis rather than a comparative study, and no statistical adjustment was made for baseline or treatment differences between cohorts. Consequently, numerically lower crude incidence rates observed in the AMGEVITA^®^ cohort should not be interpreted as evidence of reduced infection risk relative to other anti-TNF agents. Differences in baseline characteristics, including biologic experience, corticosteroid exposure, disease activity, and follow-up duration may have contributed to observed differences in crude rates. In particular, the higher proportion of prior corticosteroid use in the anti-TNF comparator cohort may have influenced infection risk. As this was a registry-based descriptive analysis without imputation of baseline covariates, missing data may have influenced characterization of patient demographics and disease features. However, safety outcomes were obtained using multiple data sources, including national administrative linkages, which reduces the likelihood of systematic under-capture of serious adverse events. Furthermore, the analyses were based on cumulative, aggregated data provided by the BSRBR-RA, and patient-level information was not available to support adjusted modeling or analyses of first events. Apparent differences in the number of reported SAEs between cohorts partly reflect the longer observation period of the anti-TNF group, which is inherent to the registry’s longitudinal design rather than an analytic decision. Despite these constraints, the use of a well-established national registry and standardized data capture supports the reliability of the findings and their relevance to real-world clinical practice. Ongoing follow-up of the AMGEVITA^®^ cohort over the duration of the PASS study is expected to provide additional person-time and event data, allowing for a more comprehensive evaluation of long-term safety outcomes. This analysis enhances the evidence base on AMGEVITA^®^ use in routine clinical practice, offering insights from a large, national registry encompassing patients with diverse comorbidities, treatment experiences, and demographic backgrounds that are often underrepresented in controlled trials. The results reinforce the utility of ongoing registry-based pharmacovigilance in characterizing the long-term safety of biosimilars and highlight opportunities for future research using patient-level and adjusted analyses to explore safety outcomes across different clinical contexts.

## Data Availability

This study used data from two sources: Amgen and the British Society for Rheumatology Biologics Register for Rheumatoid Arthritis (BSRBR-RA). Deidentified individual participant data from Amgen-sponsored clinical studies may be requested by qualified researchers. Requests are subject to review and approval and must comply with Amgen’s data sharing policies. Full details on Amgen’s data sharing procedures, including how to submit a request, are available at: (https://wwwext.amgen.com/science/clinical-trials/clinical-data-transparency-practices/clinical-trial-data-sharing-request) . Data from the BSRBR-RA are managed by the British Society for Rheumatology and are available to researchers through a formal application process. Access to these data may involve collaboration with the register team and is subject to review and associated costs for non-members. Detailed guidance on how to request BSRBR-RA data, including application deadlines and eligibility criteria, can be found at: (https://www.rheumatology.org.uk/improving-care/registers/requesting-registers-data) .
